# Elevated triglyceride-glucose (TyG) index predicts incidence of Prediabetes: a prospective cohort study in China

**DOI:** 10.1186/s12944-020-01401-9

**Published:** 2020-10-15

**Authors:** Jing Wen, Anping Wang, Guangxu Liu, Meiping Wang, Yingting Zuo, Wei Li, Qi Zhai, Yiming Mu, Herbert Y. Gaisano, Yan He, Jingtao Dou

**Affiliations:** 1 Department of Epidemiology and Biostatistics, School of Public Health, Capital Medical University, Beijing, China; 2 Department of Endocrinology, the First Medical Center, Chinese PLA General Hospital, Beijing, China; 3 Department of Medicine, University of Toronto, Toronto, Canada; 4 Municipal Key Laboratory of Clinical Epidemiology, Beijing, China

**Keywords:** Triglyceride-glucose index, Prediabetes, Impaired glucose tolerance, Lipid metabolism, Insulin resistance, Predictive value, Prospective cohort study

## Abstract

**Background:**

Prediabetes has become a pandemic. This study aimed to identify a better predictor for the incidence of prediabetes, which we hypothesize to be the triglyceride-glucose (TyG) index, a simplified insulin resistance index. We compared its predictive value with the other common risk factors of prediabetes.

**Methods:**

The participants of this analysis were derived from the Risk Evaluation of cAncers in Chinese diabeTic Individuals: a lONgitudinal (REACTION) study. A total of 4543 participants without initial prediabetes or diabetes were followed up for 3.25 years. Using multivariate logistic regression model, the associations between baseline obesity, lipid profiles and non-insulin-based insulin resistance indices with the incidence of prediabetes were analyzed. To assess which is better predictor for the incidence of prediabetes, the area under curves (AUCs) calculated from the receiver operating characteristic curve analyses were used to evaluate and compare with the predictive value of the different indices.

**Results:**

During the 3.25 years, 1071 out of the 4543 participants developed prediabetes. Using the logistic regression analysis adjusted for some potential confounders, the risk of incidence of prediabetes increased 1.38 (1.28–1.48) fold for each 1–SD increment of TyG index. The predictive ability (assessed by AUCs) of TyG index for predicting prediabetes was 0.60 (0.58–0.62), which was superior to the indices of obesity, lipid profiles and other non-insulin-based insulin resistance indices. Although the predictive ability of the TyG index was overall similar to fasting plasma glucose (FPG) (*P* = 0.4340), TyG index trended higher than FPG in females (0.62 (0.59–0.64) vs. 0.59 (0.57–0.61), *P* = 0.0872) and obese subjects (0.59 (0.57–0.62) vs. 0.57 (0.54–0.59), *P* = 0.1313). TyG index had superior predictive ability for the prediabetic phenotype with isolated impaired glucose tolerance compared with FPG (*P* <  0.05) and other indices. Furthermore, TyG index significantly improved the C statistic (0.62 (0.60–0.64)), integrated discrimination improvement (1.89% (1.44–2.33%)) and net reclassification index (28.76% (21.84–35.67%)) of conventional model in predicting prediabetes than other indices.

**Conclusions:**

TyG could be a potential predictor to identify the high risk individuals of prediabetes.

## Background

Prediabetes, an intermediate stage from normal glucose tolerance (NGT) status to type 2 diabetes mellitus (T2DM), which is a high risk stage for developing T2DM [1]. Also, prediabetes is related to increased risk of developing similar T2DM complications occurring in the eye [2], kindney [3] and cardiocerebral vascular system [4]. In Mainland China, the prevalence of prediabetes has risen from 15.5% [5] in 2008 to 35.2% in 2017, thus reaching a population of approximately 357 million Chinese with prediabetes [6]. Therefore, it is of critical importance to determine the indicators that can most efficiently predict the development of prediabetes to identify those people at higher risk of prediabetes who will be subject to closer monitoring or potential early intervention.

Insulin resistance (IR) is the key underlying pathophysiological basis in disease development from NGT to prediabetes, then to full-blown and worsening T2DM; this process may take 10–20 years of development prior to diagnosis [7, 8]. Therefore, IR is a vital indictor for predicting future development of prediabetes. Recently, non-insulin-based IR indices, including the triglyceride-glucose (TyG) index [9, 10], triglyceride to high density lipoprotein cholesterol (TG/HDL-C) ratio [11, 12] and the metabolic score for IR (Mets-IR) [13], have emerged as promising surrogate indices of IR, attributed to their convenient and ubiquitous availability; and thus the ease of calculation of these indices, which were indeed found to closely related to the IR assessed by the homeostasis models (HOMA-IR) [10–12] and euglycemic-hyperinsulinemic clamp [11, 13].

Dyslipidemia is one of vital risk factor for the progression to atherosclerotic vascular disease [14]. Dyslipidemia primarily occurs in obese individuals due to the excessive accumulation of fat in the adipose tissues [15]. Several studies have shown that metabolism of fatty acids and glucose, along with insulin sensitivity, are both influenced by nutritional and genetic factors [16, 17]. It has been well established that both obesity and dyslipidemia were considered traditional risk factors for prediabetes because these two conditions can increase peripheral tissue IR [18–21]. Furthermore, to our knowledge, there has been only one cross-sectional study that had compared the discriminative ability of obesity, lipid profiles and insulin resistance in identifying prediabetes [22]. That study [22] demonstrated that the TyG index had the best discriminative power for the diagnosis of prediabetes. However, it remains unclear whether the TyG index, as predictor for the incidence of prediabetes, is superior to other indices, including obesity, abnormal lipid profiles and other non-insulin-based IR indices.

Therefore, the aim of this cohort study was to explore the association and predictive ability of TyG index for incidence of prediabetes; and to assess its superiority over the other traditional risk factors of dysglycemia. In so doing, our study would identify a better predicator for the development of prediabetes.

## Marterials and methods

### Study design and population

The Risk Evaluation of cAncers in Chinese diabeTic Individuals: a lONgitudinal (REACTION) study [23], which is an ongoing multicenter prospective cohort study. The data for this cohort study was derived from the Pingguoyuan community of Shijingshan district, Beijing, which is a single city center that was chosen from the REACTION study. Each eligible participant aged 40 years old or older was identified and recruited using the door-to-door invitation method by the trained local community workers. Exclusion criteria for this analysis were the participants: (1) with prediabetes or diabetes at baseline; (2) had incomplete information of blood glucose parameters, anthropometric measurements and metabolic profiles; (3) missing at follow-up, and (4) new onset diabetes at follow-up.

**Fig. 1:**
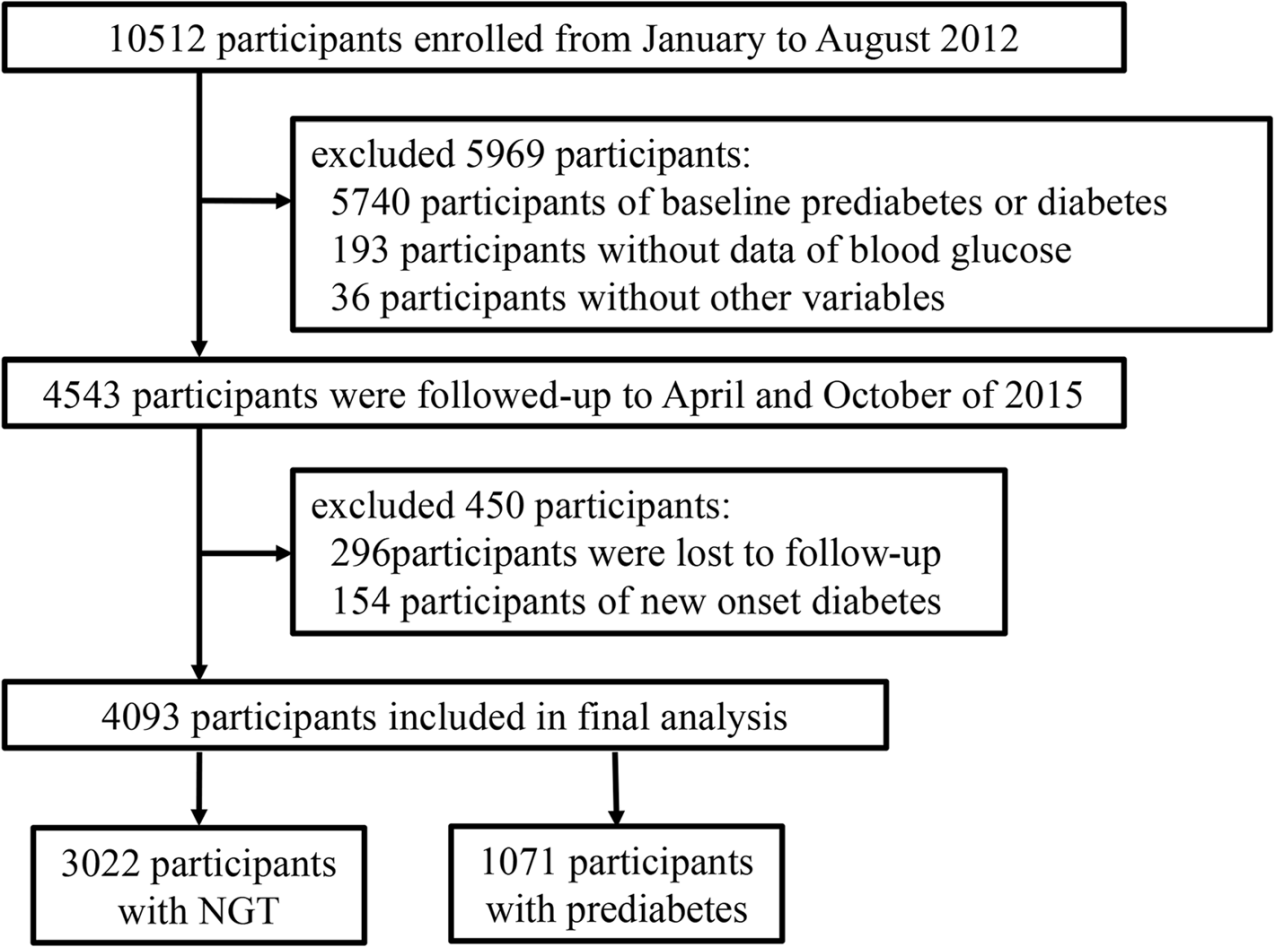
Study flow diagram

A total of 10,512 participants were enrolled from January to August 2012. Among them, 5740 of these already had prediabetes or T2DM at baseline and 229 participants had incomplete information in the questionnaire; both groups were excluded. Therefore, every one of the remaining 4543 participants were followed up between April and October 2015, a period of ~ 3.25 years. From these 4543 participants, 296 participants were missing at follow-up, giving a dropout rate of 6.52% (296 / 4543); and 154 participants were excluded because they were found to exhibit new onset of T2DM at follow-up. Finally, 4093 eligible participants wrere included in this study (Fig. 1). The REACTION study has been approved by the medical ethics committee of Ruijin Hospital (Shanghai, China). And all of the participants have provided written informed consent before taking part in this study.

### Data collection

Information on demographic characteristics (age, gender and degree of education), living habit (cigarette smoking and alcohol consumption levels), diseases history and the history of medication use were collected through the standard questionnaire by a face-to-face interview method.

Weight was measured by the electronic weight scale (Beijing Jianmin) and height was measured by the vertical height meter (Beijing Jianmin). Body mass index (BMI) was calculated as weight (kilograms) / height (meters)^2^. Waist circumference (WC) was measured between the costal margin and the iliac crest by a measuring tape. After at least five minutes of rest in the sitting position, blood pressure (BP) level was measured three times consecutively with a 1-min interval applied on the right arm by a mercury sphygmomanometer or an electronic sphygmomanometer.

Blood samples were collected after 10-h overnight fast. Serum samples were used to measure fasting plasma glucose (FPG) and lipid profiles. The 2 hour plasma glucose levels were mereasured after carring out a 75-g oral glucose tolerance test, except for the participants had a diabetes history diagnosed by a physician, who instead accepted a 100-g carbohydrate diet test.

According to cigarette smoking habit, participants were divided into current smokers, former smokers (stopped smoking more than 12 months ago) and never smokers. According to alcohol drinking habit, participants was categorized as current, former (stopped alcohol drinking more than 12 months ago) and never drinking. Education level was categorized as high school or above (higher vocational or university education) and below high school. The diagnostic criteria of hypertension was systolic blood pressure (SBP) ≥ 140 mmHg and/or diastolic blood pressure (DBP) ≥ 90 mmHg and/or the use of anti-hypertensive medicines and/or diagnosed as hypertension by a physician previously [24]. Cardiovascular disease (CVD) was defined as self-reported cerebrovascular disease and/or coronary heart disease diagnosed by a physician. The diagnostic criteria of abnormal lipid metabolism was triglycerides (TG) ≥ 2.3 mmol/L and/or total cholesterol (TC) ≥ 6.2 mmol/L and/or low density lipoprotein cholesterol (LDL-C) ≥ 4.1 mmol/L and/or high density lipoprotein cholesterol (HDL-C) < 1.0 mmol/L and/or previously diagnosed as hyperlipidemia by a physician [25]. Overweight or obesity were defined as BMI ≥ 24.0 kg/m^2^ [26].

### Calculation of non-insulin-based IR indices

The non-insulin-based IR indices of TyG index, TG/HDL-C ratio and Mets-IR were calculated by the following equations [13]:

1. TyG index = Ln [(TG (mg/dL) × FPG (mg/dL) / 2]
2. TG/HDL-C ratio = TG (mmol/L) / HDL-C (mmol/L)
3. Mets-IR = Ln [(2 * FPG (mg/dL)) + TG (mg/dL)] * BMI / Ln (HDL-C (mg/dL))

### Outcome definition

According to the criterion of American Diabetes Association (ADA) [20], these are the definitions of the following terms. Prediabetes was defined as impaired fasting glucose (IFG) and/or impaired glucose tolerance (IGT). Isolated impaired fasting glucose (i-IFG): FPG 5.6–6.9 mmol/L and postprandial 2 h-plasma glucose (2 h-PG) < 7.8 mmol/L. Isolated impaired glucose tolerance (i-IGT): FPG < 5.6 mmol/L and 2 h-PG 7.8–11.0 mmol/L. Combined impaired fasting glucose and impaired glucose tolerance (CGT): FPG 5.6–6.9 mmol/L and 2 h-PG 7.8–11.0 mmol/L. Diabetes was defined as FPG ≥ 7 mmol/L and/or 2 h-PG ≥ 11.1 mmol/L and/or using anti-diabetic medications and/or diagnosed as diabetes by a physician previously.

### Statistical analysis

Normality was tested by Kolmogorov-Smirnov (K-S) test. The data of continuous variables were described by medians and interquartile ranges for the skewed distribution. Categorical variables were expressed as frequencies and percentages. The group differences of NGT and prediabetes for continuous variables were compared with the nonparametric test, and the *χ*^2^ tests were used for categorical variables.

The associations of non-insulin-based IR indices and prediabetes were investigated with logistic regression models. Odds ratios (ORs) were standardized by using transformed observations [(observation − mean)/SD] in the models. Potential confounders were adjusted in the multivariable analyses. The predictive value of non-insulin-based IR indices for prediabetes was evaluated by the area under curves (AUCs) which calculated by the receiver operating characteristic (ROC) curve analyses. Additionally, C-statistics index, integrated discrimination improvement (IDI), net reclassification index (NRI) were performed to evaluate the improvement of predictive value of non-insulin-based IR indices beyond traditional risk factors.

All statistical analyses were performed using the SPSS software version 23.0 and R software version 3.5.0. And a *P* <  0.05 was considered statistically significant.

## Results

### Baseline characteristics of the participants

After application of the inclusion and exclusion criteria, overall 4093 participants were included in this study, finally (Fig. 1). These participants had an averaged age of 55 (50–59) years old, of which 68.8% (2818) were female. The median follow-up time was 3.25 (3.17–3.25) years. Compared to the participants with NGT, the participants with prediabetes were more likely to be older, and they had a higher proportion of family history of diabetes, history of hypertension, and higher levels of BMI, WC, SBP, DBP, TyG, TG/HDL-C and Mets-IR (Table 1).

**Table 1:** Baseline characteristics of study participants categorized by glucose metabolism status at follow-up

	Overall (*n* = 4093)	NGT (*n* = 3022)	Prediabetes (*n* = 1071)	*P*
Age(years)	55 (50–59)	54 (50–58)	55 (51–60)	< 0.001
Gender				0.962
Male	1275 (31.2)	942 (31.2)	333 (31.1)	
Female	2818 (68.8)	2080 (68.8)	738 (68.9)	
High school or above, n (%)	2679 (65.5)	1996 (66.0)	683 (63.8)	0.178
Cigarette smoking, n (%)				0.651
No smoking	3176 (77.6)	2350 (77.8)	826 (77.1)	
Quit smoking	146 (3.6)	103 (3.4)	43 (4.0)	
Current smoking	771 (18.8)	569 (18.8)	202 (18.9)	
Alcohol drinking, n (%)				0.756
No drinking	2853 (69.7)	2097 (69.4)	756 (70.6)	
Quit drinking	77 (1.9)	58 (1.9)	19 (1.8)	
Current drinking	1163 (28.4)	867 (28.7)	296 (27.6)	
Family history of diabetes, n (%)	892 (21.8)	613 (20.3)	279 (26.1)	< 0.001
BMI (kg/m^2^)	24.8 (22.8–27.0)	24.6 (22.6–26.8)	25.4 (23.2–27.4)	< 0.001
WC (cm)	81 (75–87)	81 (75–87)	83 (77–88)	< 0.001
SBP (mmHg)	126 (117–137)	125 (116–136)	129 (120–139)	< 0.001
DBP (mmHg)	74 (68–81)	74 (68–80)	75 (69–82)	< 0.001
FPG (mmol/L)	5.1 (4.9–5.3)	5.1 (4.9–5.3)	5.2 (5.0–5.4)	< 0.001
2 h-PG (mmol/L)	6.1 (5.2–6.8)	5.9 (5.1–6.6)	6.6 (5.7–7.1)	< 0.001
TG (mmol/L)	1.18 (0.85–1.65)	1.13 (0.83–1.58)	1.35 (0.95–1.86)	< 0.001
HDL-C (mmol/L)	1.44 (1.22–1.71)	1.46 (1.24–1.73)	1.39 (1.16–1.65)	< 0.001
TG/HDL-C	0.82 (0.52–1.29)	0.77 (0.50–1.22)	0.97 (0.61–1.50)	< 0.001
TyG	8.47 (8.14–8.81)	8.42 (8.11–8.76)	8.62 (8.28–8.95)	< 0.001
Mets-IR	35.22 (31.08–39.89)	34.60 (30.86–39.35)	36.63 (32.15–41.40)	< 0.001
Hypertension, n (%)	1351 (33.0)	923 (30.5)	428 (40.0)	< 0.001
CVD, n (%)	289 (7.1)	201 (6.7)	88 (8.2)	0.086

The data were expressed as median (interquartile) for skewed distributed continuous variables and numbers (percentages) for categorical variables. *P* value: from non-parametric test for skewed continuous variables and chi-square test for categorical variables. *NGT* normal glucose tolerance, *WC* waist circumference, *FPG* fasting plasma glucose, *2 h-PG* postprandial 2 h-plasma glucose, *TyG* triglyceride-glucose index, *Mets-IR* metabolic score for insulin resistance

### TyG index was the most closely associated with the incidence of prediabetes compared to the other indices

During follow-up, 1071 (26.2%) of the 4093 non-diabetic or prediabetic (NGT) participants developed prediabetes, among which the phenotype of i-IFG was 3.5% (143 / 4093), i-IGT was 20.0% (819 / 4093) and CGT was 2.7% (109 / 4093).

**Fig. 2:**
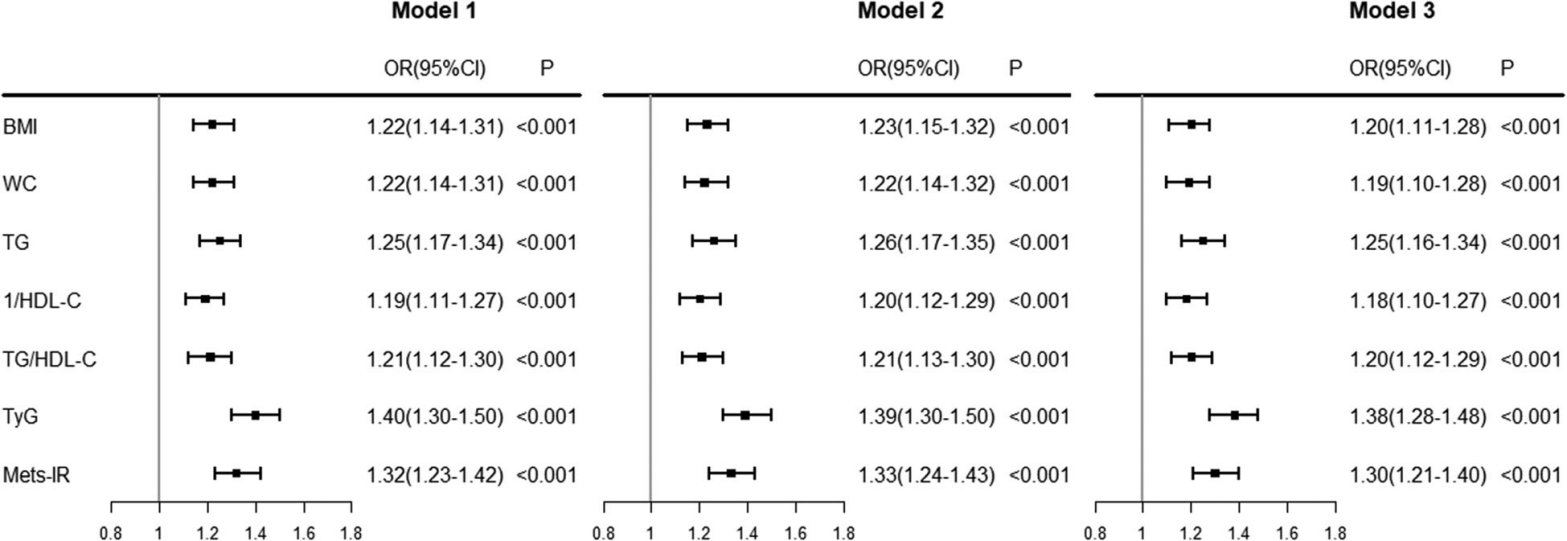
Association of indices with prediabetes. OR (odds ratio) and 95%CI (95% confidence interval): from logistic regression analysis. The reference group was NGT. Horizontal lines represent 95% CIs. Model 1: unadjusted; Model 2: adjusted for age, gender, smoking, alcohol drinking, education level and family history of diabetes; Model 3: Model 2 + adjusted for hypertension and CVD

Using logistic regression analyses and adjusted for age, cigarette smoking, alcohol drinking, education level, family history of diabetes, hypertension and history of CVD, the TyG index increased for each 1-SD, the risk of incidence of prediabetes increased by 38% (OR, 1.38 (95%CI 1.28–1.48)). TyG was the most closely associated with the incidence of prediabetes compared to the other indices examined, including TG/HDL-C, Mets-IR, TG, 1/HDL-C, BMI and WC (Fig. 2). Results from the sensitivity analyses that included the participants of new onset of T2DM (*n* = 154) as outcome with prediabetes, the association between TyG index and the incidence of prediabetes and T2DM remained similar (Table S1).

### TyG index had the highest ability to predict the incidence of prediabetes

To find a better indicator for the incidence of prediabetes, the predictive values of the variables in a ROC analysis were compared. The AUC and its 95% CI of the TyG index for prediabetes was 0.60 (0.58–0.62), which was significantly higher than almost indices assessed, except FPG (AUC = 0.59 (0.57–0.61), *P* = 0.4340) (Table 2). In the sensitivity analysis, the similar results were observed by adding the participants of new onset T2DM during the follow-up (Table S2).

**Table 2:** Performance of the indices in predicting the incidence of prediabetes

Indices	AUC (95% CI)	Cut-off point	*P*
BMI	0.561 (0.541–0.581)	25.66	0.0011
WC			
Male(*n* = 1275)	0.527 (0.489–0.564)	93.20	0.0415
Female(*n* = 2818)	0.577 (0.553–0.600)	77.50	0.0046
TG	0.591 (0.571–0.611)	1.24	< 0.0001
1/HDL-C	0.553 (0.533–0.573)	0.68	< 0.0001
TG/HDL-C	0.589 (0.569–0.609)	0.99	< 0.0001
TyG	0.600 (0.581–0.620)	8.45	Ref.
Mets-IR	0.578 (0.558–0.598)	35.08	0.0178
FPG	0.590 (0.571–0.610)	5.04	0.4340

*AUC* area under the curve. *P* value from the comparison of *AUCs*, the reference indicator was *TyG* index

Since the predictive value were quite similar for TyG index and FPG, the subgroup analyses were conducted to find out whether these two indices might differentially predict. The predictive value of the TyG index showed a trend towards being significantly higher than FPG in female (*P* = 0.0872) and obese participants (*P* = 0.1313). In the other subgroups, the predictive values of these two indices remained similar (Table 3).

**Table 3:** Performance of the TyG index versus FPG in predicting the incidence of prediabetes in subgroups with the different characteristics

Subgroup	AUC (95% CI)	Cut-off point	*P*
**Gender**			
Male (*n =* 1275)			
TyG	0.568 (0.532–0.604)	8.56	Ref.
FPG	0.595 (0.560–0.631)	5.27	0.2636
Female (*n =* 2818)			
TyG	0.616 (0.592–0.639)	8.45	Ref.
FPG	0.589 (0.566–0.613)	5.04	0.0872
**Age**			
< 60 years (*n* = 3204)			
TyG	0.606 (0.584–0.629)	8.47	Ref.
FPG	0.603 (0.581–0.625)	5.04	0.8179
≥ 60 years (*n* = 889)			
TyG	0.576 (0.535–0.617)	8.41	Ref.
FPG	0.540 (0.499–0.580)	5.07	0.1863
**Lipid metabolism**			
With normal lipid metabolism (*n* = 3041)			
TyG	0.591 (0.567–0.615)	8.45	Ref.
FPG	0.599 (0.576–0.621)	5.04	0.6223
With abnormal lipid metabolism (*n* = 1052)			
TyG	0.588 (0.551–0.624)	8.86	Ref.
FPG	0.562 (0.525–0.600)	5.05	0.3248
**Obesity status**			
18.5 ≤ BMI < 24.0 kg/m^2^ (*n* = 1581)			
TyG	0.594 (0.560–0.628)	8.46	Ref.
FPG	0.620 (0.587–0.653)	5.01	0.2635
BMI ≥ 24.0 kg/m^2^ (*n* = 2462)			
TyG	0.590 (0.566–0.615)	8.47	Ref.
FPG	0.565 (0.541–0.590)	5.04	0.1313

To compare the predictive value of the TyG index and the other indices for prediabetic phenotypes, ROC curve analyses were conducted in i-IFG, i-IGT and CGT, separately. The TyG index was found to have a superior predictive value for i-IGT compared with other indeces, including obesity, lipids profiles and FPG (Table 4).

**Table 4:** Performance of the indices in predicting the incidence of prediabetic phenotypes

Indices	AUC (95% CI)	Cut-off Point	*P*
**i-IFG (*n*** **= 143)**			
BMI	0.574 (0.526–0.621)	23.44	0.5784
WC			
Male (*n* = 1001)	0.510 (0.479–0.542)	82.00	0.2888
Female (*n* = 2146)	0.615 (0.594–0.636)	78.50	0.0858
TG	0.533 (0.483–0.584)	1.51	< 0.0001
1/HDL-C	0.571 (0.521–0.620)	0.76	0.5670
TG/HDL-C	0.551 (0.502–0.601)	1.19	0.6031
TyG	0.556 (0.507–0.606)	8.75	Ref.
Mets-IR	0.585 (0.538–0.632)	38.06	0.2296
FPG	0.716 (0.676–0.757)	5.17	< 0.0001
**i-IGT (*n*** **= 819)**			
BMI	0.549 (0.527–0.571)	25.65	0.0003
WC			
Male (*n* = 1178)	0.515 (0.486–0.544)	93.50	0.0170
Female (*n* = 2663)	0.561 (0.542–0.580)	77.50	0.0003
TG	0.592 (0.570–0.613)	1.25	< 0.0001
1/HDL-C	0.543 (0.521–0.565)	0.68	< 0.0001
TG/HDL-C	0.587 (0.565–0.609)	0.88	0.0112
TyG	0.597 (0.575–0.619)	8.45	Ref.
Mets-IR	0.565 (0.543–0.588)	34.34	0.0023
FPG	0.551 (0.529–0.573)	5.04	0.0017
**CGT (*n*** **= 109)**			
BMI	0.635 (0.580–0.690)	25.13	0.1052
WC			
Male (*n* = 980)	0.623 (0.592–0.654)	90.50	0.5833
Female (*n* = 2151)	0.660 (0.639–0.680)	83.50	0.2619
TG	0.661 (0.611–0.711)	1.24	< 0.0001
1/HDL-C	0.604 (0.549–0.658)	0.79	0.0011
TG/HDL-C	0.655 (0.605–0.706)	1.05	0.0014
TyG	0.682 (0.634–0.730)	8.45	Ref.
Mets-IR	0.666 (0.612–0.721)	38.48	0.4761
FPG	0.717 (0.677–0.757)	5.12	0.2787

*i-IFG* isolated impaired fasting glucose, *i-IGT* isolated impaired glucose tolerance, *CGT* combined impaired fasting glucose and impaired glucose tolerance

### TyG index improves the predictive ability for prediabetes of conventional risk factors

To examine whether the TyG index could improve the predictive value of the conventional risk factors of prediabetes, including age, gender, family history of diabetes, cigarette smoking, alcohol drinking, education level, hypertension and history of CVD, the C statistic, IDI and NRI were calculated (Table 5). For prediabetes as the outcome, the C statistic of the conventional model was significantly improved with the addition of TyG index (from 0.58 to 0.62, *P* <  0.0001). Furthermore, the discriminatory power and risk reclassification of the TyG index were also substantially better, showing an IDI of 1.89% (1.44–2.33%, *P* <  0.0001) and NRI of 28.76% (21.84–35.67%, *P* <  0.0001). The C statistic, discriminatory power and risk reclassification were all more improved after adding the TyG index to the conventional model compared to other indices, including TG/HDL-C, Mets-IR, TG, 1/HDL-C, BMI, WC and FPG.

**Table 5:** Improvement in predicting prediabetes by adding the indices to the conventional risk factors

Prediabetes	C statistic		IDI		NRI	
	Estimate (95% CI)	*P*	Estimate(95% CI), %	*P*	Estimate (95% CI), %	*P*
**Conventional model**	0.584 (0.564–0.603)		Ref.		Ref.	
+ BMI	0.599 (0.579–0.619)	0.0049	0.61 (0.36–0.86)	< 0.0001	17.16 (10.21–24.11)	< 0.0001
+ WC	0.598 (0.579–0.618)	0.0076	0.60 (0.35–0.85)	< 0.0001	15.46 (8.51–22.41)	< 0.0001
+ TG	0.609 (0.589–0.628)	< 0.0001	1.03 (0.68–1.38)	< 0.0001	24.70 (17.89–31.50)	< 0.0001
+ 1/HDL-C	0.599 (0.579–0.619)	0.0054	0.61 (0.35–0.86)	< 0.0001	19.76 (12.82–26.69)	< 0.0001
+ TG/HDL-C	0.603 (0.584–0.622)	< 0.0001	0.73 (0.43–1.02)	< 0.0001	25.44 (18.72–32.15)	< 0.0001
+ TyG	0.622 (0.603–0.641)	< 0.0001	1.89 (1.44–2.33)	< 0.0001	28.76 (21.84–35.67)	< 0.0001
+ Mets-IR	0.613 (0.593–0.633)	0.0001	1.35 (0.97–1.73)	< 0.0001	21.93 (15.00–28.87)	< 0.0001
+ FPG	0.620 (0.604–0.642)	< 0.0001	1.68 (1.29–2.08)	< 0.0001	27.67 (20.82–34.52)	< 0.0001

Conventional risk factors included age, gender, family history of diabetes, cigarette smoking, alcohol drinking, education level, hypertension and *CVD*

*IDI* integrated discrimination improvement, *NRI* net reclassification index

Obesity is the main risk factor for abnormal glucose metabolism. This has in part led to the ADA recommend that testing for prediabetes and/or T2DM should be considered in the overweight and obese adults with at least one additional risk factors for T2DM. Thus, the improvement of the predictive value of the TyG index versus obesity combined with those additional risk factors for prediabetes were examined. This analysis revaled that the TyG index had a superior predictive value compared with BMI when combined with any of the additional risk factors (Table 6).

**Table 6:** Improvement in predicting prediabetes by adding the TyG index versus BMI to the different conventional risk factors

Prediabetes	C statistic		IDI		NRI	
	Estimate (95% CI)	P*	Estimate (95% CI), %	*P*	Estimate (95% CI), %	*P*
**Family history of diabetes**						
+ TyG	0.606 (0.586–0.625)	0.0013	2.19 (1.72–2.67)	< 0.0001	30.57 (23.66–37.47)	< 0.0001
+ BMI	0.571 (0.551–0.591)		0.83 (0.55–1.12)	< 0.0001	18.98 (12.03–25.92)	< 0.0001
**CVD**						
+ TyG	0.602 (0.583–0.622)	0.0014	2.19 (1.71–2.65)	< 0.0001	30.70 (23.80–37.60)	< 0.0001
+ BMI	0.565 (0.545–0.585)		0.79 (0.51–1.07)	< 0.0001	18.27 (11.33–25.22)	< 0.0001
**Hypertension**						
+ TyG	0.610 (0.590–0.629)	0.0005	1.47 (0.95–2.00)	< 0.0001	17.82 (10.87–24.77)	< 0.0001
+ BMI	0.577 (0.557–0.597)		0.55 (0.31–0.79)	< 0.0001	14.02 (7.06–20.97)	< 0.0001
**1/HDL-C**						
+ TyG	0.600 (0.580–0.620)	0.0017	1.60 (1.19–2.00)	< 0.0001	22.30 (15.37–29.23)	< 0.0001
+ BMI	0.567 (0.547–0.587)		0.50 (0.28–0.72)	< 0.0001	17.46 (10.51–24.41)	< 0.0001
**TG**						
+ TyG	0.603 (0.584–0.623)	0.0364	1.34 (0.97–1.71)	< 0.0001	26.93 (20.19–33.66)	< 0.0001
+ BMI	0.588 (0.568–0.607)		0.50 (0.27–0.72)	< 0.0001	13.66 (6.70–20.61)	< 0.0001

*IDI* integrated discrimination improvement, *NRI* net reclassification index. * Comparison of the C statistic between *TyG* and *BMI* plus one of conventional risk factor

## Discussion

These are the major points of this cohort study. First, the TyG index had better predictive ability for the incidence of prediabetes than the obesity indices of BMI and WC, lipid profiles of TG and HDL-C, and non-insulin-based insulin resistance indices of TG/HDL-C and Mets-IR. Second, TyG index’s predictive value was similar to FPG, with a trend towards being superior to FPG in female and obese subjects. And the TyG index had a superior predictive ability than FPG for the prediabetic phenotype of i-IGT. Third, when combined with some conventional risk factors, the TyG index’ predictive ability for the incidence of prediabetes was significantly improved over BMI. These results all indicate that the TyG index should be considered to be a potential and reliable predictor for the incidence of prediabetes for broad clinical usage. Furthermore, this was the first prospective cohort study that compared the predictive value for prediabetes among its common risk factors of obesity, lipid profiles and insulin resistance.

Obesity is an important risk factor for prediabetes [27]. Several previous population studies had demonstrated that obesity to be associated with prediabetes. A cross-sectional study from Bangladesh that included 2293 rural adults showed that obesity was associated with IFG and IGT, and the predictive values (AUC and 95%CI) of BMI for identifying IFG and IGT were 0.61 (0.56–0.66) and 0.65 (0.60–0.69), respectively [28]. A cohort study from Singapore that included 1137 participants demonstrated that BMI increase with each 1–unit increased the risk of incidence of prediabetes by 6% (Risk ratio (95%CI): 1.06 (1.02–1.09)) and obesity (BMI ≥ 27.5 kg/m^2^) was related to the increased risk of prediabetes by 61% (Risk ratio (95%CI): 1.61 (1.11–2.36)) [29]. Our study has convinclgly illustrated that TyG index was more closely related to the incidence of prediabetes compared with BMI (OR (95%CI): 1.30 (1.21–1.40) vs. 1.20 (1.11–1.28)) and had a better predictive ability (AUC and (95%CI): 0.60 (0.58–0.62) vs. 0.56 (0.54–0.58), *P* = 0.0011). To further compare the predictive ability of the TyG index versus BMI, their improvement to the model with traditional risk factors of abnormal glucose metabolism were examined by calculating the C statistics, IDI and NRI; these results demonstrated that the TyG index effectively improved the predictive ability of the model that was better than BMI.

To our knowledge, the ADA guideline for diabetes is the only one that clearly addressed the screening criteria for prediabetes. The guideline recommended that testing for prediabetes and/or T2DM should be conducted at those are overweight or obesity and have at least one risk factor of diabetes [20]. To assess whether the TyG index could be a suitable alternative indicator to BMI in predicting prediabetes, the predictive ability of the TyG index versus BMI was compared by adding each to some of the common risk factors recommended by the ADA guideline for prediabetes, including first-degree relative with diabetes, history of CVD and hypertension, elevated HDL-C and TG level; and the results demonstrated that the predictive abilities were all significantly improved by replacing BMI with the TyG index (Table 6). These results led us to conclude that the TyG index tended to be superior to BMI in predicting the incidence of prediabetes.

Insulin resistance and dyslipidemia are important risk factors for prediabetes. Our study demonstrated that TyG index’s predictive ability was also higher than these two sets of indices, including lipid profiles of TG and HDL-C, and indices of non-insulin-based insulin resistance of TG/HDL-C and Mets-IR. These results also demonstrated that TyG index had similar predictive value compared with FPG, but trended towards being superior to FPG in female and obese subjects. Taken together, TyG may be a more suitable predictor than FPG for predicting prediabetes in female and obese subjects.

Prediabetes, including three phenotypes of i-IFG, i-IGT and CGT; among them, i-IGT, was the most common phenotype that accounted for a remarkable 65.3% of prediabetes cases in the Chinese population [5]. The Da Qing study performed in 577 Chinese adults with IGT demonstrated that the cumulative incidence of T2DM in 6 years was 67.7% [30]. A meta-analysis demonstrated that IGT was most correlated with the increased all-cause cardiovascular mortality [4]. It’s critical to identify the high risk population that has IGT. However, it is not easy to detect IGT in routine clinical practice. In this cohort study, the results showed that the TyG index was a superior predictor for i-IGT compared with FPG and other indices, which could identify the high risk population of i-IGT at the early time.

Several previous studies have provided clues to understand the underlying mechanisms. First, several studies demonstrated that the TyG index may have strong predictive ability for IR [31–33]. And the TyG index had a high sensitivity of 96.5% to diagnose IR compared with the hyperinsulinemic-euglycemic clamp in Mexican population [9]. Two validation studies conducted on a Brazilian and a Korean population demonstrated the TyG index had a superior performance compared with HOMA-IR in predicting IR [31, 32]. Since IR is a major pathophysiological basis of dysglycemia, this may explain the reason for TyG index having a higher predictive ability for prediabetes. Second, TyG is a composite index of fasting TG and FPG. Glycerol and fatty acids are two products of TG lipolysis can enhance liver gluconeogenesis [34, 35]. This may also be a reason for why TyG to be more potent in predicting prediabetes than FPG in female and obese subjects, who usually have higher TG levels. Third, oxidative stress and inflammation could lead to and predict IR, both able to significantly increase the HOMA-IR and TyG index levels [36, 37]; this may also explain the correlation of the TyG index and prediabetes.

### Study strength and limitations

This cohort study has some major strengths. Firstly, the association and predictive value of non-insulin-based IR indices, obesity and lipid profiles for prediabetes were compared simultaneously; these are most common risk factors for prediabetes. Second, prediabetes was defined as the impairment of FPG and 2 h-PG in this study; therefore, IGT, a major prediabetes phenotype in Chinese people, was not ignored. Third, this prospective cohort study enabled us to judge the appropriate order of exposure and outcome. Finally, a series of sensitivity analyses was performed to increase the rigor of our study and found consistent findings for the participants that exhibited new onset of prediabetes and T2DM. Nevertheless, there remained the following limitations in our study. First, the study participants only had an average follow-up time of 3.25 years, which could be considered to be relatively short. Second, the study participants were only followed up for one time, and the information were obtained at only two time points of baseline and follow-up visits. Third, there has no available data of serum insulin levels and the predictive value of TyG index and HOMA-IR couldn’t be compared; and there was also no available data on the antihyperlipidemia drug use, which may influence the results to a certain degree.

## Conclusions

The risk of subjects with prediabetes progressing to T2DM was high. Therefore, earlier identification and intervention of subjects at high risk of prediabetes may delay or even prevent the development of prediabetes and subsequent T2DM and its ensuing complications. Our study demonstrated that the TyG index is a superior indicator to predict prediabetes and is so conveniently accessible; and thus, the TyG index may be considered for wide clinical practice as a potential indicator for predicting the incidence of prediabetes. Notably, IGT was difficult to detect in routine clinical practice, therefore the individuals at high risk for IGT could be identified earlier by using the TyG index as a more reliable predictor to further delay the development of T2DM and its complication.

## Supplementary information

**Additional file 1: Table S1.** Sensitivity analysis of the association of indices with prediabetes and diabetes. **Table S2.** Sensitivity analysis of the performance of indices to predict the incidence of prediabetes and diabetes.

## Data Availability

The datasets used to support this study are not freely available in view of participants’ privacy protection.

## Abbreviations

TyG: Triglyceride-glucose
REACTION: Risk evaluation of cAncers in chinese diabetic individuals: a longitudinal
AUC: Area under curve
ROC: Receiver operating characteristic curve
WC: Waist circumference
Mets-IR: Metabolic score for insulin resistance
FPG: Fasting plasma glucose
IDI: Integrated discrimination improvement
NRI: Net reclassification index
NGT: Normal glucose tolerance
T2DM: Type 2 diabetes mellitus
IR: Insulin resistance
ADA: American Diabetes Association
HOMA-IR: Homeostasis model assessment of IR
TG/HDL-C: Triglyceride to high-density lipoprotein cholesterol
BMI: Body mass index
SBP: Systolic blood pressure
DBP: Diastolic blood pressure
CVD: Cardiovascular disease
TG: Triglycerides
TC: Total cholesterol
LDL-C: Low density lipoprotein cholesterol
HDL-C: High density lipoprotein cholesterol
IFG: Impaired fasting glucose
IGT: Impaired glucose tolerance
i-IFG: Isolated impaired fasting glucose
i-IGT: Isolated impaired glucose tolerance
CGT: Combined impaired fasting glucose and impaired glucose tolerance
ORs: Odds ratios
