# Supplementary material for: Elevated triglyceride-glucose (TyG) index predicts incidence of Prediabetes: a prospective cohort study in China
Source: Lipids Health Dis. 2020 Oct 15;19:226. doi: 10.1186/s12944-020-01401-9 (PMC7565371; doi:10.1186/s12944-020-01401-9)
Supplement: Supplementary file 1 — Additional file 1: Table S1. Sensitivity analysis of the association of indices with prediabetes and diabetes. Table S2. Sensitivity analysis of the performance of indices to predict the incidence of prediabetes and diabetes. [file 12944_2020_1401_MOESM1_ESM.docx]

**Table S1** Sensitivity analysis of the association of indices with prediabetes and diabetes

| Indices | Model 1 | |  | Model 2 | |  | Model 3 | |
| --- | --- | --- | --- | --- | --- | --- | --- | --- |
|  | OR (95% CI) | *P* |  | OR (95% CI) | *P* |  | OR (95% CI) | *P* |
| Overall (n = 4247) |  |  |  |  |  |  |  |  |
| BMI | 1.26 (1.17–1.34) | < 0.001 |  | 1.26 (1.17–1.34) | < 0.001 |  | 1.22 (1.14–1.30) | < 0.001 |
| WC | 1.26 (1.18–1.35) | < 0.001 |  | 1.26 (1.17–1.35) | < 0.001 |  | 1.22 (1.13–1.31) | < 0.001 |
| TG | 1.25 (1.17–1.34) | < 0.001 |  | 1.25 (1.17–1.34) | < 0.001 |  | 1.24 (1.16–1.33) | < 0.001 |
| 1/HDL-C | 1.19 (1.11–1.27) | < 0.001 |  | 1.19 (1.11–1.28) | < 0.001 |  | 1.17 (1.09–1.26) | < 0.001 |
| TG/HDL-C | 1.20 (1.12–1.29) | < 0.001 |  | 1.20 (1.12–1.29) | < 0.001 |  | 1.19 (1.11–1.28) | < 0.001 |
| TyG | 1.40 (1.31–1.49) | < 0.001 |  | 1.39 (1.30–1.49) | < 0.001 |  | 1.37 (1.28–1.47) | < 0.001 |
| Mets-IR | 1.34 (1.26–1.43) | < 0.001 |  | 1.35 (1.26–1.44) | < 0.001 |  | 1.31 (1.22–1.40) | < 0.001 |

*OR* (odds ratio) and 95% CI (95% confidence interval): from logistic regression analysis. The reference group was *NGT*.

Model 1: unadjusted;

Model 2: adjusted for age, gender, cigarette smoking, alcohol drinking, education level and family history of diabetes;

Model 3: Model 2 + adjusted for hypertension and *CVD*.

**Table S2** Sensitivity analysis of the performance of indices to predict the incidence of prediabetes and diabetes

| Indices | AUC (95% CI) | Cut-off Point | Sensitivity | Specificity | *P* |
| --- | --- | --- | --- | --- | --- |
| Overall (n = 4247) |  |  |  |  |  |
| BMI | 0.565 (0.546–0.584) | 25.66 | 47.7 (44.8–50.5) | 64.4 (62.6–66.1) | 0.0022 |
| WC |  |  |  |  |  |
| Male | 0.541 (0.505–0.576) | 88.80 | 44.2 (39.2–49.4) | 64.0 (60.9–67.1) | 0.0261 |
| Female | 0.563 (0.542–0.583) | 80.70 | 56.8 (53.8–59.8) | 52.3 (50.4–54.2) | 0.0010 |
| TG | 0.591 (0.572–0.610) | 1.24 | 56.7 (53.8–59.5) | 58.2 (56.4–59.9) | < 0.0001 |
| 1/HDL-C | 0.553 (0.533–0.572) | 0.68 | 58.8 (56.0–61.5) | 49.7 (47.9–51.5) | < 0.0001 |
| TG/HDL-C | 0.589 (0.571–0.608) | 0.76 | 64.7 (62.0–67.4) | 49.6 (47.8–51.4) | 0.0015 |
| TyG | 0.600 (0.582–0.619) | 8.45 | 63.2 (60.4–65.9) | 52.7 (50.9–54.5) | Ref. |
| Mets-IR | 0.583 (0.564–0.602) | 35.08 | 60.7 (57.9–63.5) | 52.6 (50.8–54.4) | 0.0418 |
| FPG | 0.591 (0.572–0.609) | 5.04 | 70.1 (67.5–72.7) | 45.9 (44.1–47.7) | 0.4343 |

*AUC* area under the curve. *P* value from the comparison of *AUC*s, the reference indicator was *TyG* index.
